# Psychosocial and Cognitive Dimensions of Aging and Cancer: Communication, Resilience, and Health Challenges

**DOI:** 10.1093/geroni/igaf122.757

**Published:** 2025-12-31

**Authors:** Sean Halpin

## Abstract

In this symposium, we explore the psychosocial and cognitive dimensions of aging and cancer, focusing on how humor, communication, and psychological well-being shape patient experiences across different health contexts, including cancer and other chronic conditions. We bring together diverse methodological approaches to examine the complexities of navigating health challenges as an older adult. First, Halpin will present on the intersection of cognitive load and socio-emotional adaptation theory in patient education for older adults with multiple myeloma. He will focus on how information delivery strategies affect patient understanding and emotional adaptation. Next, Hardt will discuss older adults’ experiences of depression during cancer. Using qualitative methods, she describes older adults’ views of depression during cancer and strategies for coping with cancer, including mental health services. Chidebe will then examine access to psychotherapy, psychological support and psycho-oncology concerns among older cancer patients, using quantitative methods to explore the psychological challenges faced by older adults with cancer and potential avenues for mental health support. Finally, Krok-Schoen will explore the relationship between physical functioning, self-efficacy, and quality of life in cancer survivors, drawing on quantitative data to assess key psychosocial factors influencing survivorship. By integrating our perspectives, we provide a broader understanding of how psychosocial adaptation, humor, and cognitive processing intersect in aging populations facing complex health challenges. Our discussion will offer practical applications for improving communication, patient education, and psychosocial support strategies for older adults. Cancer and Aging Interest Group Sponsored Symposium

